# Clients Mixed Experiences of Receiving Job Support and Getting a Job When Participating in Individual Placement and Support in Norway

**DOI:** 10.1192/j.eurpsy.2025.763

**Published:** 2025-08-26

**Authors:** L. G. Kinn, L. Davidson, K. J. Oedegaard, E. Langeland

**Affiliations:** 1 Department of Welfare and Participation, Western Norway University of Applied Sciences, Bergen, Norway; 2 School of Medicine, Yale University, New Haven, United States; 3 Department of Clinical Medicine, University of Bergen; 4 Department of Health and Caring Sciences, Western Norway University of Applied Sciences, Bergen, Norway

## Abstract

**Introduction:**

People with severe mental illness (SMI) view employment as central to their recovery process, principally when work task is experienced as meaningful, manageable, and comprehensible. However, unemployment rates remain extremely high among people with SMI, especially those diagnosed with schizophrenia. The costs are high: significant numbers of people are at risk of loss of life’s purpose, social isolation, poverty, and even suicide. Internationally, there is a knowledge gap of lived experience of receiving IPS in a job development and working phase. We need to know more about those who do not get or stay in a job. Correspondingly, in a national context more knowledge is needed about mainstream ESs’ practices from an IPS client’s perspective.

**Objectives:**

Thus, the aim of this study was to explore clients’ experiences of receiving job support from employment specialists (ESs) working with individual placement and support (IPS) in Norway. IPS is developed to help people with severe mental illness (SMI) into competitive employment as an integral component of mental health services.

**Methods:**

Using a hermeneutic phenomenological methodology, this study comprises individual semi-structured interviews with ten participants engaged in IPS at two districts psychiatric centers. Data analysis was conducted according to systematic text condensation.

**Results:**

Three themes emerged: (1) ES—a door opener? (2) Striving to sidestep a “spider web” of triggers at and away from work; and (3) Calling for a safer route.

**Conclusions:**

This study highlights the importance of ESs offering IPS clients’ opportunities to try out diverse jobs and focusing more on assessing the work environment in the jobs they place people into. Our findings imply that ESs should spend more time on building a good working alliance with both clients and employers, and pay more attention on understanding individuals’ vocational capacities and support needs at the worksite. The ES training should focus not simply on the technical processes of job development and placement, but more directly on empowering clients to stay focused on their vocational ambitions and prospects. The salutogenic model of health can help ESs to analyze whether clients experience workplaces as meaningful, manageable, and comprehensible.

**Disclosure of Interest:**

None Declared

